# Combining Federated Machine Learning and Qualitative Methods to Investigate Novel Pediatric Asthma Subtypes: Protocol for a Mixed Methods Study

**DOI:** 10.2196/57981

**Published:** 2024-07-08

**Authors:** Jie Xu, Sankalp Talankar, Jinqian Pan, Ira Harmon, Yonghui Wu, David A Fedele, Jennifer Brailsford, Jennifer Noel Fishe

**Affiliations:** 1 Department of Health Outcomes and Biomedical Informatics University of Florida College of Medicine Gainesville, FL United States; 2 Center for Data Solutions University of Florida College of Medicine - Jacksonville Jacksonville, FL United States; 3 Department of Clinical and Health Psychology University of Florida College of Public Health and Health Professions Gainesville, FL United States; 4 Department of Emergency Medicine Center for Data Solutions University of Florida College of Medicine - Jacksonville Jacksonville, FL United States

**Keywords:** pediatric asthma, machine learning, federated learning, qualitative research

## Abstract

**Background:**

Pediatric asthma is a heterogeneous disease; however, current characterizations of its subtypes are limited. Machine learning (ML) methods are well-suited for identifying subtypes. In particular, deep neural networks can learn patient representations by leveraging longitudinal information captured in electronic health records (EHRs) while considering future outcomes. However, the traditional approach for subtype analysis requires large amounts of EHR data, which may contain protected health information causing potential concerns regarding patient privacy. Federated learning is the key technology to address privacy concerns while preserving the accuracy and performance of ML algorithms. Federated learning could enable multisite development and implementation of ML algorithms to facilitate the translation of artificial intelligence into clinical practice.

**Objective:**

The aim of this study is to develop a research protocol for implementation of federated ML across a large clinical research network to identify and discover pediatric asthma subtypes and their progression over time.

**Methods:**

This mixed methods study uses data and clinicians from the OneFlorida+ clinical research network, which is a large regional network covering linked and longitudinal patient-level real-world data (RWD) of over 20 million patients from Florida, Georgia, and Alabama in the United States. To characterize the subtypes, we will use OneFlorida+ data from 2011 to 2023 and develop a research-grade pediatric asthma computable phenotype and clinical natural language processing pipeline to identify pediatric patients with asthma aged 2-18 years. We will then apply federated learning to characterize pediatric asthma subtypes and their temporal progression. Using the Promoting Action on Research Implementation in Health Services framework, we will conduct focus groups with practicing pediatric asthma clinicians within the OneFlorida+ network to investigate the clinical utility of the subtypes. With a user-centered design, we will create prototypes to visualize the subtypes in the EHR to best assist with the clinical management of children with asthma.

**Results:**

OneFlorida+ data from 2011 to 2023 have been collected for 411,628 patients aged 2-18 years along with 11,156,148 clinical notes. We expect to complete the computable phenotyping within the first year of the project, followed by subtyping during the second and third years, and then will perform the focus groups and establish the user-centered design in the fourth and fifth years of the project.

**Conclusions:**

Pediatric asthma subtypes incorporating RWD from diverse populations could improve patient outcomes by moving the field closer to precision pediatric asthma care. Our privacy-preserving federated learning methodology and qualitative implementation work will address several challenges of applying ML to large, multicenter RWD data.

**International Registered Report Identifier (IRRID):**

DERR1-10.2196/57981

## Introduction

Globally, nearly 22 million children are diagnosed with asthma [1]. In the United States alone, 4.5 million children are living with asthma, and exacerbations from asthma account for an estimated 500,000 emergency department visits and 64,000 hospitalizations annually [2-4]. Care for school-aged children with asthma accounts for US $5.92 billion of US health care spending [5]. Asthma is a chronic respiratory disease characterized by constriction of the lower airways, resulting in wheezing, cough, and shortness of breath [1]. Although asthma can be diagnosed with pulmonary function tests, it is often diagnosed clinically by a physician based on patient history and examination [1].

Pediatric asthma is a heterogeneous disease characterized by a range of etiologies, triggers, clinical manifestations, severities, and treatment responses [6]. Such disease heterogeneity can be classified into subphenotypes or subtypes. However, current pediatric asthma subtypes are primarily confined to allergic versus nonallergic asthma [7]. This simple dichotomous classification does not account for overlapping subtypes, the evolution of subtypes over time as a child grows and develops, differences in severity (especially considering racial and ethnic disparities), or the influence of social determinants of health (SDOH; defined as nonmedical factors that influence health outcomes according to the World Health Organization [8]). It is imperative to improve subtype characterization of pediatric asthma to facilitate more personalized and effective primary and emergency care and reduce the burden of care at both individual and population levels.

Machine learning (ML) methods such as deep neural networks that capture longitudinal information to learn new patient representations, combined with downstream clustering, are well-suited for identifying relevant subtypes [9]. Clustering algorithms for pediatric asthma subtypes would ideally be applied to large data sets containing voluminous electronic health record (EHR) data, including free-text note data, from diverse areas and populations. However, the inclusion of protected health information (PHI) and other sensitive data in EHR free-text notes raises privacy concerns, which requires the deidentification of structured and narrative EHR information. Manual deidentification is time-consuming and cannot be scaled up to large-scale studies, whereas automated deidentification of EHRs using machines cannot completely remove PHI. Federated learning is a subfield of ML that can address these privacy issues by allowing a central server to communicate with local sites to learn a global model, the parameters of which are then sent back to the local sites [10].

In this study, we propose to apply novel, privacy-preserving federated ML methods to identify and model pediatric asthma subtypes and their progression over time [11]. Federated learning is an ML technique that trains a shared global model with a central server while keeping data at the local sites, as opposed to aggregating individual site data together [10]. We will apply federated learning to a large, distributed clinical research network containing nearly 20 million demographically and socioeconomically diverse patients from the southeastern United States (Florida, Georgia, and Alabama). Despite the increasing sophistication of various ML techniques, there remain significant challenges in implementing ML algorithms, decision tools, and other forms of clinical support in frontline health care settings [12]. Therefore, we will also interweave our technical data science methods with qualitative implementation science research to “design for dissemination” [13], and ultimately optimize the translation of our research findings into clinical practice.

## Methods

### Overview of the Data Source and Study Design

This study uses the OneFlorida+ clinical research network [14], which is a large regional network covering linked and longitudinal patient-level real-world data (RWD) from over 20 million patients from Florida, Georgia, and Alabama in the United States. OneFlorida+ contains detailed patient demographic data, diagnoses, procedures, vital signs, medications, and laboratory results from Medicaid and Medicare claims; vital statistics; and EHRs from 15 clinical partners. Data quality in OneFlorida+ is maintained at each step of the data pipeline, with quality assurance governed by the OneFlorida+ data trust team.

**Figure 1:**
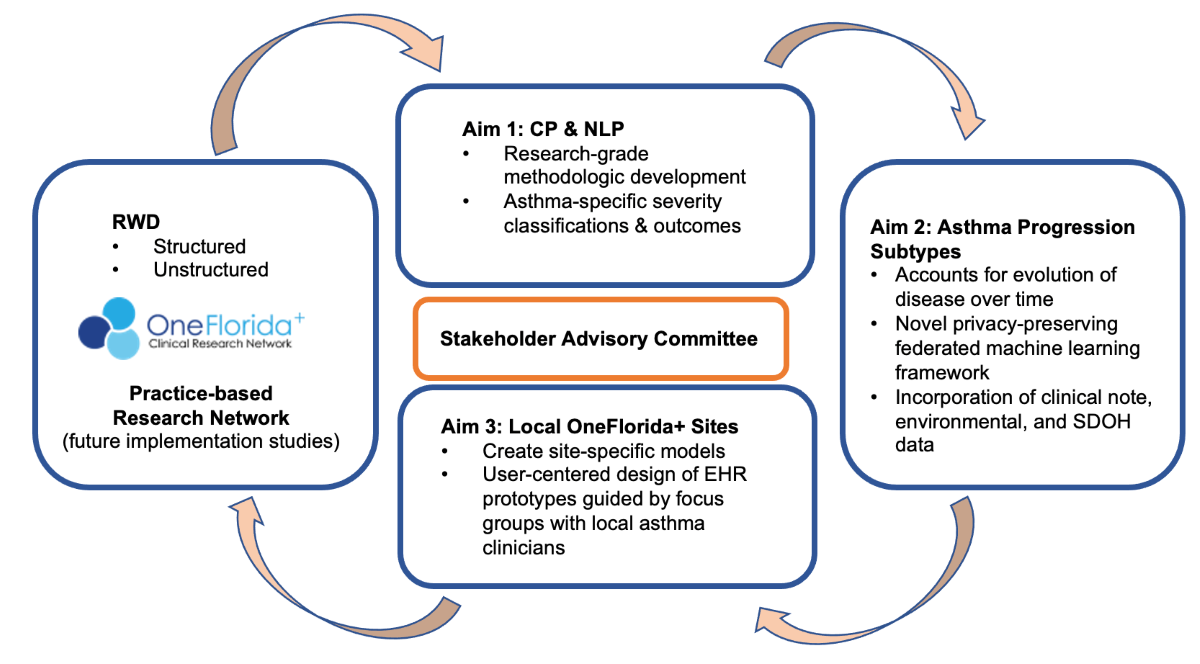
Overview of the mixed methods study design. CP: computable phenotype; EHR: electronic health record; NLP: natural language processing; RWD: real-world data; SDOH: social determinants of health.

To accomplish our study aims (Figure 1), we will initially develop and optimize a research-grade pediatric asthma computable phenotype (CP) and a clinical natural language processing (NLP) pipeline to accurately extract pediatric asthma–relevant information from EHRs. Subsequently, we will use deep learning models to capture the temporal representation of patients at each local site. Through federated learning, we will collaboratively learn the representation models across different sites and leverage federated clustering to characterize harmonized pediatric asthma subtypes and their progression across the sites in the OneFlorida+ database. We will also integrate focus groups with OneFlorida+ clinical network asthma clinicians to assess the clinical utility of the identified subtypes. Additionally, we will develop initial EHR prototypes for visualizing subtype information and evaluate the utility of these prototypes.

### Ethical Considerations

The University of Florida Institutional Review Board (IRB) approved the subtyping study, including the use of both structured and unstructured data with a waiver of informed consent (UF IRB#202002779). For each participating site in this study, data will be securely stored on Health Insurance Portability and Accountability Act–compliant servers approved for the storage of PHI. Access to these files will be restricted to individuals included in the IRB protocol, ensuring compliance with ethical and regulatory standards. Specifically, for the University of Florida site, data will also be stored on HiPerGator for the required computing resources. We will adhere to the established procedures outlined in the “PHI on HiPerGator Process” documentation [15], which includes registering the project through a request in the University of Florida’s Integrated Risk Management system and ensuring all project members sign the corresponding agreement form. For the focus group study, we will consent participants and compensate them with US $150 per session.

### Stakeholder Advisory Committee

We will initially establish a stakeholder advisory committee to provide guidance on the study design, interpretation of results, and the dissemination and implementation of findings. In addition to the core study team, we will include multiple pediatric asthma stakeholders, including patients and caregivers, in the advisory committee (Textbox 1). The committee will meet twice yearly for the 5-year duration of the study. Meetings will comprise study team presentations on study progress and results. Committee members will be prompted for specific feedback and will also have ample opportunities to provide any input and suggestions to the study team. The number of members, their professional roles, and the guidance requested from the committee were determined according to Patient-Centered Outcomes Research Institute Research Engagement Principles [16].

**Composition of the stakeholder advisory committee.**

- Patients and caregivers (n=2 teenage patients and n=2 caregivers)
- School nurses (n=1 elementary school, n=1 middle/high school)
- Clinical providers (n=2 primary care pediatricians, n=1 emergency or intensive care pediatric physician, n=1 allergist, n=1 pediatric pulmonologist)
- Health systems administrator (n=1)
- Public health agency representative (n=1)
- Health care insurance representative (n=1)
- Core study team (n=4)

### Developing a Pediatric Asthma CP

Accurately identifying pediatric patients with asthma from retrospective EHRs is crucial for ensuring the fidelity of the entire project. Although diagnostic criteria for asthma exist, as published by the National Asthma Education and Prevention Program [17], many clinicians rely on combinations of patient history, physical exam findings, and/or diagnostic tests to diagnose asthma in children. Additionally, pediatric asthma can present with varied and subtle symptoms. Therefore, simple inclusion and exclusion criteria based on *International Classification of Diseases* codes may not be sufficiently nuanced or comprehensive to identify all pediatric patients with asthma.

CPs are clinical states determined solely from EHR and/or other data that can be processed by a computer [18]. To date, there has been no widely validated and accepted pediatric asthma CP developed. Existing CPs for identifying pediatric patients with asthma from longitudinal EHR data (eg, CAPriCORN, PheKB, and NLP-PAC [19-21]) have shown variable performance when externally validated. We will refine these existing CPs and, if necessary, develop our own de novo pediatric asthma CP using a variety of structured and unstructured data available in the University of Florida and OneFlorida+ EHRs. If the development of new CPs is required, we will apply ML-based models (eg, transformer-based models). We will use standard CP development methods, including chart review by expert physicians to label charts. According to the methods of Buderer et al [22], given a Type I error of .05 and an acceptable width of the 95% CI of 0.1 with an estimated prevalence of pediatric asthma in the United States of 8.1% [1], for a minimum specificity and sensitivity of 0.9, we will need to manually review at least 427 charts as the minimum sample size. Due to sample size requirements for the NLP pipeline (see below), we will review 500 charts in the qualifying pediatric age range of 2-18 years. We plan to sample the charts from the entire pediatric data set to avoid bias in the development of the CP. We may need to review more charts to have a sufficient number of asthma cases.

### Developing a Clinical NLP Pipeline to Categorize Pediatric Patients With Asthma and Their Disease Severity

A key aspect of pediatric asthma research using RWD is appropriately identifying patients with asthma and categorizing them by severity, clinical outcomes, and other characteristics (eg, SDOH). However, relying solely on structured data limits the ability of algorithms to identify and classify pediatric patients with asthma [23]. For example, some symptoms defining asthma severity (eg, nighttime cough, frequency of rescue inhaler usage) may be found only in clinical notes. Therefore, incorporating unstructured data using NLP can enhance the performance of pediatric asthma CPs [24]. We will extract patient characteristics from clinical narratives (ie, clinical concept extraction/named-entity recognition) by systematically examining different NLP models, with a focus on deep learning models (especially transformers such as Bidirectional Encoder Representations from Transformers), which have shown superior performance to other model types [25]. These models scan input words in sequence and determine the optimal labeling based on context features from surrounding words.

To build these models, we will perform a comprehensive literature search for keywords and phrases related to history, symptoms, environmental factors, and SDOH pertinent to pediatric asthma. We will then highlight these keywords and phrases in notes using the web-based annotation system BRAT [26]. The coprincipal investigator, who is a clinician experienced in the treatment of pediatric asthma, will train a team of 5 research coordinators to annotate records. At least 2 annotators will annotate the same 500 notes to develop an annotated gold-standard data set. Any disagreements will be resolved by the clinical coprincipal investigator. We will then split this data set into training and testing data sets, and the corpus will be normalized using a pipeline previously constructed for sentence boundary detection and tokenization [27]. We will apply our large language model GatorTron [28] to handle both negation and abbreviations in a unified model. Model performance (ie, how accurately the model extracted the entities of intent) will be evaluated on the testing data set with the microaveraged precision, recall, and *F_1_*-score using strict and lenient (ie, partial matching of boundary) criteria. The best-performing model will be used to extract relevant pediatric asthma data from clinical notes, which can be incorporated into refining the CPs mentioned above.

Of note, with both CP and NLP pipeline development, the same data can exist in both structured and unstructured forms, which may not have the same value, making data harmonization a key consideration for this project. To select the most reliable and detailed information source, we will carefully review all pediatric asthma variables and CP rules during each project step for redundancy, accuracy, and complimentary/discordant data. For example, a medication prescription can be found in the structured data (eg, inhaled corticosteroid), but the true frequency of its use by the patient may be found in the clinical notes (eg, once daily instead of twice daily). To mitigate redundancy, we will use strategies such as data deduplication [29], feature selection [30], and integrating insights from both structured and unstructured sources [31]. To enhance accuracy and reconcile any discrepancies between data sets, we will use techniques such as data fusion [32] and expert review.

### Modeling Pediatric Asthma Subtypes and Their Temporal Progression Pathways With a Patient Representation Learning Model

After identifying pediatric patients with asthma using the most accurate CP, we will model the temporal representation of the asthma severity levels. Toward this end, we will include 2 years of data before and a minimum of 2 years after asthma symptoms first appear, as data prior to an asthma diagnosis allows for the inclusion of antecedent data that may be critical to asthma subtyping. We will define the asthma onset date by the first date in which the patient is classified as a positive case as per the most accurate pediatric asthma CP.

**Figure 2:**
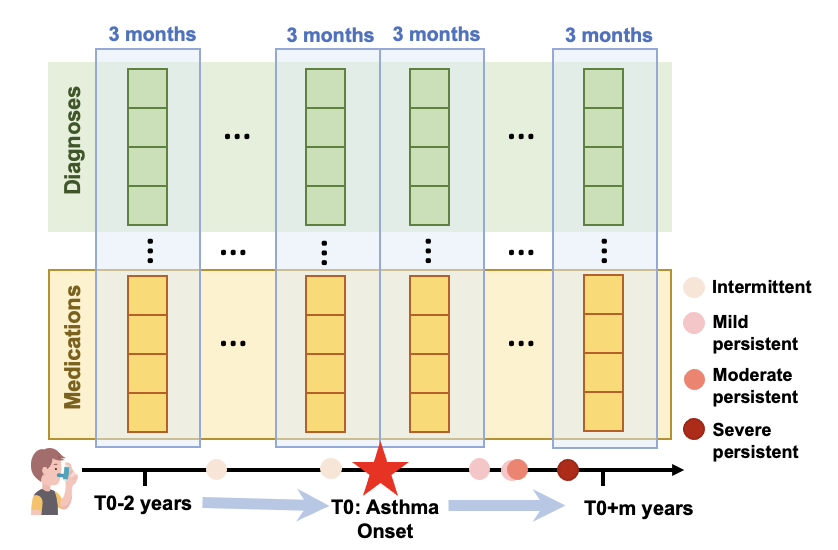
The asthma temporal trajectory in electronic health records.

As illustrated in Figure 2, we will aggregate relevant EHR data for each patient into vectors within 3-month blocks (ie, window sizes, although we will consider varying window sizes in this project). Each vector corresponds to a particular event type (eg, clinical encounter, diagnosis, procedure, medication, or symptoms) based on discrete structured data and data extracted from clinical notes using NLP. For example, the dimensionality of a diagnosis vector equals the number of distinct diagnosis codes, with each dimension’s value representing the frequency of that code appearing in the current 3-month period. We will divide each patient’s data into multiple subsequences. We plan to explore a wide range of deep learning methods, including long short-term memory autoencoders (baseline) [33], outcome-oriented transformers [34], and block-recurrent transformers [35], based on our initial review of state-of-the-art models to effectively represent the sequential records of pediatric patients with asthma.

**Figure 3:**
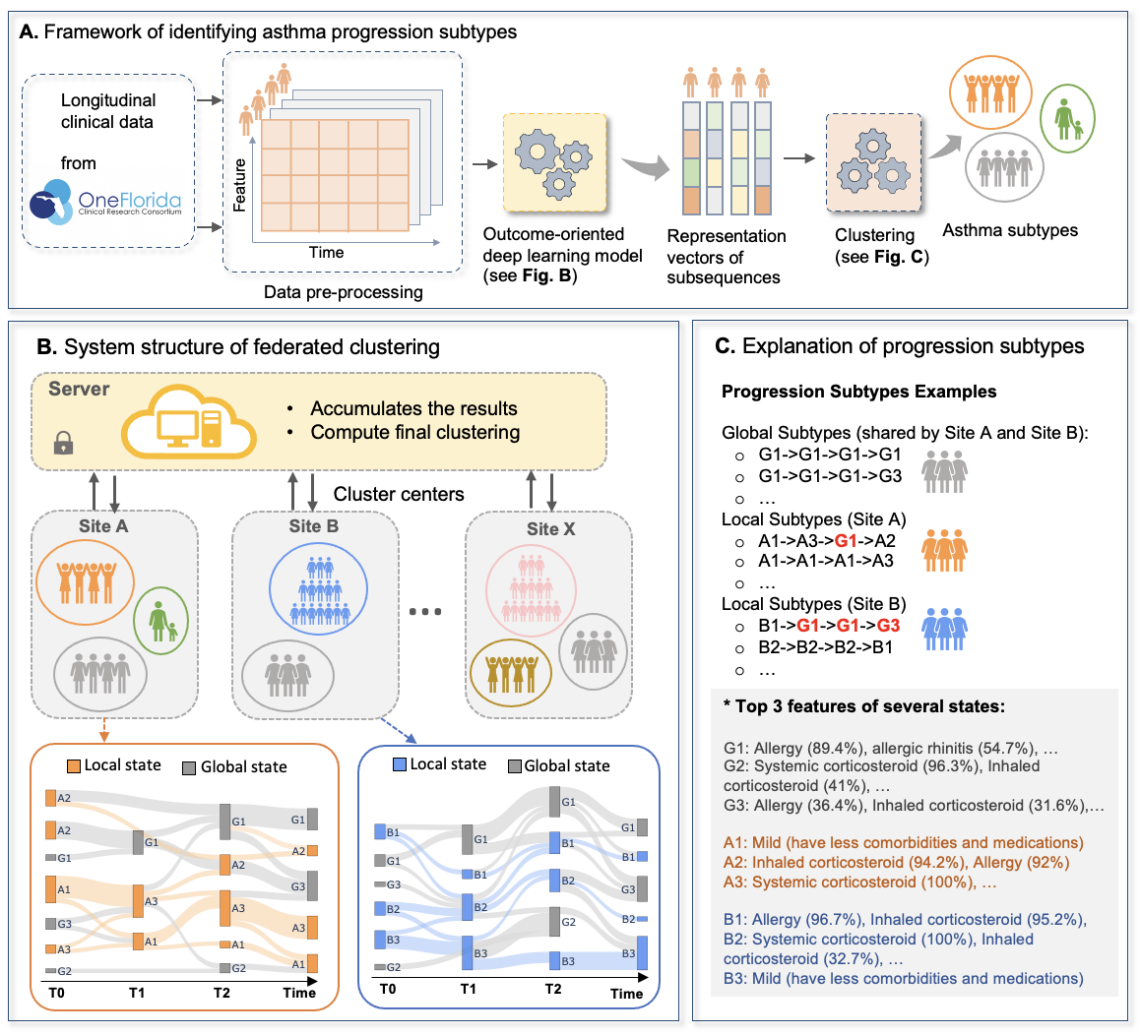
Identifying asthma progression subtypes. (A) Framework of identifying asthma progression subtypes. (B) System structure of federated clustering. (C) Explanation of progression subtypes.

After obtaining temporal representations, we will apply hierarchical agglomerative clustering [36] to determine the clusters of the subsequences (ie, states), as illustrated in Figure 3. Subsequences will be grouped according to the similarity of their progression embedding vectors learned through the previous modeling process. We will select the Ward method for hierarchical agglomerative clustering [36]. Unlike other methods that measure distance directly, the Ward method focuses on analyzing the variance of clusters. It achieves this by iteratively merging the most similar clusters, with the goal of minimizing the increase in the error sum of squares upon cluster combination. By reducing variance within each cluster, the Ward method facilitates the formation of compact and distinct clusters. Once we have established the clusters of subsequences (ie, states), we will determine the states for each patient based on the cluster centers of the corresponding subsequences derived from that patient. The trajectory pattern of the patient is represented by different states that change over time (ie, the progression from one state to another). Each progression subtype will include patients with similar trajectory patterns. For instance, if a patient is divided into four subsequences and three states (eg, A1, A2, A3) are identified using the clustering algorithm [10], the trajectory pattern of that patient would be “A1 to A3 to A1 to A2.” Additionally, we will develop predictive models to identify key features that transition patients from one state to another, using different states as labels.

### Federated Learning for Identifying Asthma Progression Subtypes Across OneFlorida+ Networks

In the subsection above, we described how we will model pediatric asthma subtypes and their temporal progression pathways using a patient representation learning model within individual local institutions. However, extending this approach to distributed clinical research networks such as OneFlorida+ necessitates careful consideration of privacy issues. Hence, we propose leveraging federated learning, which is a technique that connects fragmented data sources to learn a global model without sharing sensitive patient data across sites [10]. Under this federated learning framework, each OneFlorida+ site periodically communicates the local updates to a central server. The central server then aggregates these updates and sends back the parameters of the updated global model to the sites. This process ensures that patient data remain decentralized and secure while allowing collaborative model training across distributed sites.

In addition, we will use a federated clustering approach to identify OneFlorida+ site–specific subtypes and shared subtypes across all OneFlorida+ sites, accounting for the heterogeneity of patients with asthma across different health care systems. Figure 3B shows examples of site-specific progression subtypes for two theoretical sites (ie, site A and site B). The colorful progressions (ie, orange paths and blue paths at the bottom of Figure 3B) are local site-specific progression subtypes. The grey paths represent shared progression subtypes (ie, created after federated learning clustering identified global states by leveraging other sites’ data).

### Studying the Utility and Integration of Progression Subtype Data Into Clinical Practice

While asthma severity classifications are currently used as clinical decision support for pediatric asthma clinicians [37], our subtype data are expected to encompass a broader range of features. Prior to integrating our findings into a user-centered design of an EHR-based clinical decision support system, it is essential to know how clinicians will use the novel asthma progression subtypes generated by this project, specifically what information is most relevant (eg, subtype characteristics, progression patterns, modifiable risk factors) and how it is used in different practice settings (eg, primary care, subspecialty care, emergency/inpatient care).

Therefore, we have adopted a “design for dissemination” approach [13], and will use the Promoting Action on Research Implementation in Health Services (PARIHS) framework [38] to interweave our quantitative subtyping results with qualitative feedback from pediatric asthma clinicians. We will conduct sequential rounds of focus groups to better understand the clinical utility of this information and plan for its eventual clinical implementation. The first round of focus groups will involve practicing pediatric asthma clinicians from local OneFlorida+ sites, where we will present the progression subtype results (including both site-specific and global model data). We will recruit focus group participants from the spectrum of clinicians who care for children with asthma to understand how progression subtype data are interpreted and used across the continuum of care, including primary care physicians (eg, pediatricians, family medicine practitioners), emergency physicians, pediatric inpatient hospitalists and critical care physicians, pediatric pulmonologists, and allergists, as well as health information technology professionals. For clinician participants, we will recruit MDs, DOs, and other allied health professionals who autonomously see patients and have been in practice at least 2 years.

We will deductively code focus group transcripts using the PARIHS framework, which was selected owing to its ability to systematically explore themes key to the implementation of research into clinical practice (Table 1). Two members of the study team trained in qualitative methods will separately code the transcripts. We will calculate Cohen κ for coder interrater reliability and resolve discrepancies by study team consensus. Using NVivo, we will combine related codes to construct overarching themes and relate those themes to PARIHS elements and subelements (Table 1).

**Table 1:** Promoting Action on Research Implementation in Health Services (PARIHS) framework and themes [38] to explore in the first round of focus groups.

PARIHS element			Relation to study
**Evidence**			
	Research	Subtype features, number, progressions	
	Clinical and patient experience	SDOH^a^, health disparities, patient age, health literacy	
	Local data	Site-specific versus global subtype models	
**Context**			
	Culture	Clinician, health care system, patient/caregiver, community	
	Leadership	Health care system and payer attitudes, clinician incentives	
	Evaluation	Asthma severity classification, patient-centered outcomes	
**Facilitation**			
	Purpose	Clinical decisions, risk factor modification	
	Role	Specialty, clinical setting	
	Skills and attributes	Specialty, clinical setting, EHR^b^ fluency	

^a^SDOH: social determinants of health.

^b^EHR: electronic health record.

Based on feedback from the first round of focus groups and our stakeholder advisory committee, we will develop multiple EHR design prototypes, according to a user-centered design, for incorporating pediatric asthma progression subtype data (considering both shared/global subtypes and site-specific subtypes). Subsequently, we will conduct a second round of focus groups with the same participants to present these EHR design prototypes in a high-fidelity manner (ie, demonstration video showing how the system is integrated into the clinicians’ EHR with functionality, and, when applicable, a mock-up system where the end users can interact in a simulated EHR environment). During these sessions, we will gather clinicians’ feedback on how they would interpret the information and interact with alerts and other functions (when applicable), identify usability issues, and solicit preferences and suggestions for improvement.

### Study Limitations and Potential Expansions

While OneFlorida+ is a large sample of pediatric patients with asthma, it does not represent all pediatric patients with asthma, and thus our subtypes may not be generalizable nationwide or worldwide. Missing data is possible, particularly with regard to patient-reported variables. When possible, we will query the free-text data to fill in missing variables. For variables likely to be missing at random, we will use tools such as multiple imputation by chained equations and regression-based imputations [39]. For variables missing not at random, we will consider selection model–based methods, including outcome-dependent sampling for longitudinal outcomes [39]. We will leverage the infrastructure of OneFlorida+ and our study team’s existing relationships with clinical partners at each identified site to recruit practicing clinicians for focus groups. In the unlikely event that we do not meet our target recruitment for focus group participants, we can expand the focus groups beyond the OneFlorida+ network.

## Results

### Funding Acquisition and Recruitment

This study obtained funding from the National Institutes of Health/National Heart, Lung, and Blood Institute on September 1, 2023 (1R01HL169277). In September 2024, we began data abstraction. The OneFlorida+ data trust contains approximately 21.29 million patients. Between 2011 and 2023, OneFlorida+ recorded data for 411,628 patients aged 2-18 years and contained 11,156,148 clinical notes.

### Current Asthma CPs

As an initial step in developing pediatric asthma CPs, we conducted a rapid review of the published English-language literature for existing CPs for pediatric asthma. The review identified four CPs for pediatric asthma and a fifth for classifying pediatric asthma severity (Table 2).

**Table 2:** Existing pediatric asthma computable phenotypes (CPs), data composition, and performance metrics.

CP	Structured data	Unstructured data	Performance metrics^a^			
			PPV^b^ (%)	NPV^c^ (%)	Sensitivity (%)	Specificity (%)
CAPriCORN [40]	Yes	No	90	96	89	96
PheKB [41]	Yes	Yes	67	90	73	87
NLP-PAC [24]	Yes	Yes	89	97	92	96
Problem List [42]	Yes	No	98	N/R^d^	N/R	N/R
Pediatric Asthma Severity^e^ [43]	Yes	Yes	N/A^f^	N/A	N/A	N/A

^a^Performance metrics based off original studies.

^b^PPV: positive predictive value.

^c^NPV: negative predictive value.

^d^N/R: not reported.

^e^Performance judged by various components of computable phenotypes and combinations of components agreement with physician expert review of severity as judged by the weighted κ value, which ranged from –0.11 to 0.46.

^f^N/A: not applicable.

### Project Timeline

In the first year of the project, we will construct and optimize the pediatric asthma CP and develop a clinical NLP pipeline to better categorize pediatric asthma patients and their disease severity. In the second and third years, we will implement federated learning strategies to model pediatric asthma subtypes and their progression. In the fourth and fifth years, we will engage in focus groups with frontline asthma clinicians to assess the clinical utility of the subtypes, design EHR prototypes for clinicians to visualize subtype information, and conduct another round of focus groups to gather feedback on the EHR prototypes. Our stakeholder advisory committee will convene biannually throughout the project’s duration. We expect to publish our subtyping results in year 4 and the focus group and EHR prototype work at the end of year 5.

## Discussion

The results of this project will advance both methodologic and clinical science. With regard to technical and methodologic development, the CP and NLP pipeline can assist other pediatric asthma researchers. Our novel privacy-preserving federated learning methodology addresses several challenges associated with analyzing large multicenter RWD and provides a generalizable framework for other clinical research networks. Additionally, the framework of our federated learning methodology could also be applied to the study of subtypes of other chronic, heterogeneous diseases. Clinically, pediatric asthma progression subtypes incorporating RWD can help improve patient outcomes by moving the field closer to precision pediatric asthma care, tailoring medications, addressing potentially preventable risk factors, and preventing exacerbations that risk morbidity and mortality. Importantly, our concomitant qualitative research and stakeholder engagement lays the foundation for efficient and timely implementation of our subtypes into clinical practice.

## Appendix

Multimedia Appendix 1 Peer review report from the Center for Scientific Review Special Emphasis Panel (National Institutes of Health, USA).

## Abbreviations

CP: computable phenotype
EHR: electronic health record
IRB: Institutional Review Board
ML: machine learning
NLP: natural language processing
PARIHS: Promoting Action on Research Implementation in Health Services
PHI: protected health information
RWD: real-world data
SDOH: social determinants of health

## Data Availability

As OneFlorida+ is a data set containing identifying information, we will not make our data publicly available. We will develop tutorials and a manual of operations for the computable phenotype and natural language processing code and tools we will develop in this project. Final code will be made available as open-source software.
