# Supplementary material for: Combining Federated Machine Learning and Qualitative Methods to Investigate Novel Pediatric Asthma Subtypes: Protocol for a Mixed Methods Study
Source: JMIR Res Protoc. 2024 Jul 8;13:e57981. doi: 10.2196/57981 (PMC11263892; doi:10.2196/57981)
Supplement: Multimedia Appendix 1 [file resprot_v13i1e57981_app1.pdf]

1R01HL169277-01 Fishe, Jennifer

EARLY STAGE INVESTIGATOR
NEW INVESTIGATOR
REVISED

RESUME AND SUMMARY OF DISCUSSION: This application proposes to apply privacy-preserving machine learning tools to a large real-world data source to identify pediatric asthma subtypes and their progression over time. The project's high significance lies in its ability to advance what is currently known about the real-world implementation of asthma subtypes, though the panel noted concerns with the rigor of the prior research, as the application did not clearly state hypotheses on expected progression subtypes and how those subtypes could inform clinical care. This is a highly successful investigative team that is compatible and would ensure the success of the project. The project was considered moderately innovative, with reviewers citing concerns about the introduction of unnecessary risk by using federated learning on a centralized dataset. The panel identified several strengths of the application during the discussion, including the large, robust database of clinical notes, the stakeholder advisory committee, and the clear and strong emphasis on implementation. The panel also noted some weaknesses with the approach, including concerns related to slightly interdependent aims, the emphasis on site-specific subtypes in the federated learning vs. implementation science contexts, and the prematurity of Aim 3. There was general agreement that the potential significance of the proposed work is moderate to high, as it is not clear if it would yield results to guide clinical practice.

DESCRIPTION (provided by applicant): Asthma affects nearly 6 million children in the United States, and on average, each child with asthma experiences at least one exacerbation per year. Pediatric asthma accounts for over 790,000 emergency department visits, 64,000 hospitalizations, and nearly $6 billion in direct healthcare costs annually. Asthma disproportionately affects minority children, who are at risk for more severe outcomes. Asthma is a heterogeneous disease with a range of etiologies, triggers, severities, and treatment responses (i.e., subtypes). Despite that well-known heterogeneity, asthma subtypes are largely confined to a simple dichotomous classification of allergic versus non-allergic, which does not account for overlapping subtypes, subtype evolution, severity, nor do they include social determinants of health (SDOH). As such, if we are to reduce the burden of asthma at both the individual and population level, we must improve asthma subtype characterization to help clinicians craft more personalized primary and emergency care. To date, however, asthma subtyping studies have been limited by small sample sizes, ignored temporal information, and/or focused on individual or a handful of sites. The proliferation of large clinical research networks (CRNs) with real-world data (RWD) from electronic health records (EHRs), combined with advancements in machine learning offer unique opportunities to improve subtyping of pediatric asthma patients. Our study team's preliminary analysis of asthma exacerbations in the OneFlorida+ CRN using only structured data found five pediatric asthma subtypes which varied by race/ethnicity, severity, digital biomarkers, and comorbidities. Our work supports that there is further heterogeneity in pediatric asthma beyond the classically defined subtypes of allergic vs non-allergic. In this project, we will leverage the OneFlorida+ CRN's large repository of RWD (covering nearly 20 million patients in the southeast) and a novel privacy-preserving federated machine learning-based framework to: (1) identify pediatric asthma patients, their severity, subtypes, and disease progression (i.e., progression subtypes), and (2) fine-tune those global models to local OneFlorida+ sites with site-specific data to account for between-site heterogeneity. In addition to structured EHR data, we will include spatiotemporally linked environmental data and use natural language processing to include clinical note data such as symptoms and SDOH. To guide our work and inform implementation efforts, we will establish a stakeholder advisory committee with pediatric asthma, healthcare system, and public health stakeholders, and conduct focus groups with local OneFlorida+ site clinicians to develop and test EHR prototypes that integrate subtype

data. Pediatric asthma progression subtypes built using RWD from diverse populations combined with stakeholder engagement will move the field closer to precision primary and emergency care that improves outcomes. Our novel privacy-preserving federated machine learning methods address several challenges of RWD analysis and will be a generalizable framework for other CRNs to adopt, facilitating widespread dissemination of this work, and paving a path forward for progression subtype analyses of other chronic diseases.

PUBLIC HEALTH RELEVANCE: Pediatric asthma is a heterogeneous disease, with different etiologies, triggers, clinical manifestations, severities, and treatment responses. Identifying groups of children with asthma by those specific characteristics (i.e., asthma subtypes) can improve asthma management, including primary care and emergency treatment. This project applies novel privacy-preserving machine learning tools to a large (~ 20 million patients) real-world data source to identify pediatric asthma subtypes and their progression over time (i.e., progression subtypes), with the goal of improving care and outcomes for the millions of children with asthma in the United States.

CRITIQUE 1

Significance: 2
Investigator(s): 2
Innovation: 2
Approach: 5
Environment: 1

Overall Impact: The proposed study aims to utilize data-driven, computational approaches to identify progression subtypes for pediatric asthma and implement them in clinics. The significance of the project is high, as identification and implementation of novel and pragmatic subtypes can inform and improve clinical decision making. A major strength is to utilize innovative computational analyses and machine learning to characterize each patient with a rich representation learned from electronic health records (EHR) and computable phenotypes. There is a clear and meaningful plan to implement data-driven findings in clinics under the supervision of a stakeholder advisory committee. A very strong team of investigators will implement computational approaches to identify computable phenotypes of pediatric asthma from structured and unstructured clinical data/records. The team will also utilize data-driven approaches to identify progression subtypes of asthma and develop implementation plans. There are multiple moderate and minor weaknesses, including a lack of clear hypotheses on how identified subtypes would be useful in clinical decision making, innovative yet unnecessary use of federated learning on a centralized dataset, strong interdependence of aims, and several ambiguities in the application of the methods. Despite these weaknesses, the project is likely to have a moderate-to-high impact on the field by introducing novel ways to study asthma subtypes and their real-life implementation.

1. Significance:

Strengths

- Accurate identification of pediatric asthma subtypes by understanding risk factors and possible progression trajectories could significantly improve clinical decision making and primary care.
- The proposed study will combine structured (EHR) and unstructured data (natural language processing/NLP on clinical notes) to provide a very rich clinical presentation of pediatric asthma, including social determinants of health (SDOH) and possible outcomes.

- The findings (subtypes) will be implemented in EHR following robust pipelines (validations, discussions with stakeholders, focus groups, etc.).

Weaknesses

- This is a data-driven and unsupervised study. While investigators considered several validation steps and continuous discussions with the stakeholder advisory committee (SAC), the lack of clear hypotheses slightly diminishes the potential impact of the proposed project. Inclusion of hypotheses on expected trajectories (progression subtypes), as well as how those trajectories could possibly inform clinical care would strengthen the proposal.

2. Investigator(s):

Strengths

- This is a strong team blending expertise in pediatric/emergency asthma, computational analysis (generic machine learning, NLP, federated learning), and implementation science.
- There is a strong track record of relevant publications and studies.
- There is also a strong track record of prior collaboration.

Weaknesses

- Team will include multiple data analysts and software programmers but a single postdoctoral researcher to implement all computational models. There is a minor concern that a single postdoc may not have enough expertise to implement various methods (NLP, federated learning, clustering, etc.).

3. Innovation:

Strengths

- This is a highly innovative proposal including use of advanced machine learning models to study temporal progression (trajectories) from time series data.
- Use of CP and NLP will provide a rich characterization of patients and the clinical course of pediatric asthma, including clinical, environmental, and SDOH risk factors.
- Federated learning (FL) provides a principled way to combine data from multiple institutions without confidentiality concerns and without actual data-sharing. Developed model will be shared as an open-source project and thus can be used by other researchers as well.
- Asthma subtypes will be defined based on progression (temporal change), not snapshot characteristics, which would potentially improve its implementation value. That is, findings may provide actually useful information for clinicians (what courses are possible for this patient, how should I treat this patient?).

Weaknesses

- While use of FL is innovative and open-source code will be useful for other researchers, the main dataset is not a distributed one. That is, use of FL is not necessary for the success of the project at all; it even introduces unnecessary risk factors. Thus, one of the main novelties is omittable.

4. Approach:

Strengths

- Inclusion of SAC is very important and increases the confidence in the proposal's success.
- The plans for implementing findings in EHR are very comprehensive and well-thought.
- Proposal includes several advanced machine learning components and all of them are described/justified well (despite some ambiguities; see below), and they are suitable for the data.
- Proposed methodologies are suitable for the problem (identification of progression subtypes).
- Almost all components are unsupervised, thereby hard to validate; however, investigators propose use of several annotations, chart reviews, and validations by experts that mitigate validation concerns.

Weaknesses

- Although investigators state in the proposal that aims are not dependent, I think they are actually highly dependent. The main novelty and significance of the study is inclusion of unstructured data via NLP. If Aim 1 fails, despite presence of current CPs, Aim 2 and 3 will lose the richness provided by the proposed CPs in Aim 1. Although they have a preliminary work related to Aim 2, it is for “snapshot” subtypes, not for progression subtypes. Thus, if Aim 2 fails, they cannot implement progression subtypes in Aim 3.
- Use of unnecessary FL with a centralized dataset introduces a risk for the success and increases dependence of Aims 2 and 3.
- It is not clear why there is an emphasis on “site-specific” subtypes. In several places, I felt like there is a confusion between “site-specific” in the context of FL, and “site-specific” in the context of implementation science. Within the context of FL, site-specific would simply mean partial, inaccurate, possibly biased, and limited. FL, by combining information from all sites, addresses these issues in site-specific datasets and generates more reliable, unbiased, and complete representations. In other words, there is no point in going back to site-specific representations and findings, once you have the global ones. There is a value in site-specific representations within the context of implementation science, when the phrase “site-specific” means capturing representations and associated outcomes that are specific to certain demographics, etiologies, ethnicities, etc. But this is not the same as the phrase “site-specific” in FL terminology. Specific models (in the context of implementation science) should be constructed using global (comprehensive/inclusive) representations of FL, by studying how those global representations are differentially manifested in a specific group of people.
- It is not clear how different types of data (diagnostic codes/frequencies, medication history, SDOH, severity, etc.) will be combined in the models. What kind, if any, of normalization/scaling is considered?
- The pediatric asthma patients will be identified using EHR queries and NLP techniques, thus it is not clear how many patients will be available. Nevertheless, Table 2 provides exact numbers as if those numbers are already known.
- It is not clear how missing time points (3-month chunks) will be handled. Patients may be missing data points before and after asthma onset. I am not sure use of data imputation (e.g., MICE) would be sufficient since each time point is a multivariate instance.
- Patients will have different number time points (3-month chunks). How will the outcome-oriented long short-term memory (LSTM) model handle such differences? From Figure 4b, it seems that separate LSTM encoders will be trained for different time points, thus, this issue can be handled by varying the number of LSTM encoders used, but this should be clarified in the text. How

memory mechanism will be modeled with varying number of time points (plus, possibly missing time points).

- I assume that the representation vectors learned by the LSTM encoder will be representation of the entire time series data, not a single time point. In other words, learned representations will encode progression, not individual time points. Thus, it is not clear how such a representation, which already encodes progression, will be used in the subsequent clustering to learn individual states. To learn states, you need a representation that encodes individual time point data.

5. Environment:

Strengths

- Environment is good to complete the project. PIs have all the things needed including HPC cluster to complete the work.

Weaknesses

- None noted by reviewer.

Study Timeline:

Strengths

- None noted by reviewer.

Weaknesses

- None noted by reviewer.

Protections for Human Subjects:

Acceptable Risks and/or Adequate Protections

- This is a secondary analysis of existing data, and there is no more than minimal risk.

Data and Safety Monitoring Plan (Applicable for Clinical Trials Only):

Acceptable

- Secondary data analysis of existing data. The dataset already has safety monitoring in place.

Inclusion Plans:

- Sex/Gender: Distribution justified scientifically.
- Race/Ethnicity: Distribution justified scientifically.
- For NIH-Defined Phase III trials, Plans for valid design and analysis:
- Inclusion/Exclusion Based on Age: Distribution justified scientifically.
- The study specifically includes children as they aim to investigate pediatric asthma.
- Demographic distributions are due to composition of the existing dataset.

Vertebrate Animals:

Not Applicable (No Vertebrate Animals)

Biohazards:
Not Applicable (No Biohazards)

Resource Sharing Plans:
Acceptable

Budget and Period of Support:
Recommend as Requested

CRITIQUE 2

Significance: 4
Investigator(s): 1
Innovation: 1
Approach: 5
Environment: 2

Overall Impact: This proposal seeks to characterize new subtypes of pediatric asthma, including factors such as race/ethnicity, severity of symptoms, digital biomarkers, comorbidities, and progression. They propose doing so with a large database of real-world data from a clinical research network, creating clinic specific subtype descriptions. They will use structured electronic health record (EHR) data, spatiotemporal data, and free text notes using natural language processing (NLP). They will use an innovative federated data model to pool data across a large network of providers. They propose including stakeholder engagement in order to help clinicians craft more personalized, precision primary and emergency department (ED) care. Strengths include: importance of pediatric asthma; potential impact on the field for improved identification of pediatric asthma patients and identification of asthma subtypes for precision-medicine interventions based on non-genetic modifiable factors; applicability of federated data model learnings and resources to other diseases and datasets; innovative use of EHR data and NLP to create computable phenotypes (CPs) and federated data models; intellectually diverse and complementary team with track record in using the proposed methods; and a robust large dataset with existing infrastructure for NLP and federated data model. Weaknesses include Aim 3 assumes that the data generated from Aims 1 and 2 will inform a clinical decision support (CDS) tool, to be developed and refined through focus groups, that will substantially change clinical practice. However, it is unclear how the CDS tool will inform clinical practice, since there are no guidelines developed to inform clinical practice based on the progression subtypes, nor clear evidence-based interventions (pharmaceutical or non-pharmaceutical) that clinicians will know to reach for. A stakeholder advisory committee (SAC) will not be an adequate solution to this lack of an evidence-base. It is unclear whether the social determinants and race and ethnicity should or should not be included in the CP and progression sub-type models, with a concern that implicit bias may be baked into the models, deepening health inequities. A rationale for the timeline for the data used in the progression subtype analysis is not given, and the timeline for data availability will have substantial implications for the development of CDS tool, as some (most?) patients may not have data available in the system for two years before and two years after asthma symptom onset. Unstructured clinical notes have high risk of data missing not at random. Considering both the strengths and weaknesses of the proposal, the application’s overall impact is moderate to high.

1. Significance

Strengths

- Pediatric asthma is a highly prevalent disease with substantial morbidity (ED visits, hospitalizations).
- Defining computable phenotypes using clinical NLP would improve on current standard methods for identifying and classifying asthmatics (ICD-based definitions mostly, and some EHR definitions using only structured elements). This has the potential to more effectively identify asthmatics and severity of their disease compared to the current standard. This would meaningfully advance the field if accurate and reliable.
- Defining progression subtypes might allow for new therapeutics or more targeted therapeutics or treatment plans if subsequent clinical trials were able to use the subtypes to test new approaches.
- The learnings from the federated data model will likely be applicable to other diseases and diagnoses, which will move the field forward methodologically and can have impact in other diseases.

Weaknesses

- Early identification of subtypes would be unlikely to lead to radical transformation of clinical practice in the short term, as the proposal suggests, since clinicians would not have treatment guidelines based on subtypes and therefore would not know how to change practice. Even the more explicit and actionable potential examples given in Figure 6 do not necessarily have strong evidence bases for how to address them (e.g., obesity, difficulties with transportation). A SAC will not be an adequate solution to this lack of an evidence-base. The emphasis in Aim 3 in understanding stakeholder responses to the CDS tool seem premature. Additional preliminary data or clearer articulation of the evidence-based actionability of the progression subtypes would strengthen the rationale for Aim 3.
- It is unclear how the social determinants and race and ethnicity should or should not be included in the CP and progression sub-type models. The AAP is moving strongly away from race-based medicine (see Wright et al. Pediatrics July 2022 “Eliminating race-based medicine”). There is debate in multiple fields (e.g., epidemiology, quality measurement and value-based purchasing programs, clinical risk or prediction scores, and more), as to whether or not to include social determinants or race/ethnicity. There is a risk of baking existing bias, reflected through variable documentation in the medical record, into the algorithms. For instance, if there is differential elicitation or documentation of asthma severity in the medical record by race/ethnicity or SES, this could bias the computable phenotype or progressive subtype analysis. Or if a clinician differentially documents by race concern about a parent’s ability to care for a child with asthma due to the clinician’s implicit bias and this gets captured in the algorithm, there is a potential for deepening health inequities, if those systematically biased perceptions get perpetuated through the CP or the progression subtyping.
- It is unclear how this will inform clinical practice, as the grant suggests as the next step, due to timing of data availability and lack of clarity regarding how and when the CDS tool is generated in clinical practice. For instance, if a patient is new to the system, they will not have digital biomarkers in the EHR nor prior data to use to assess progression subtype. For all patients, would data for the CDS be generated in real time? Or only for patients who have adequate data in the system? How often would the CDS tool be regenerated since some of the data elements that go into the progression subtype might change?

- The rationale for doing the site-based analyses is not clear. What is the impact of doing them? Would different sites manage their patients differently according to their site-based progression subtypes? Methodologically, it would seem to risk over-fitting and potentially sparse data sets for the lower volume sites in particular.

2. Investigator(s)

Strengths

- Diverse team with broad range of expertise and necessarily skills to carry out this ambitious project.
- Fisce has training in implementation science as well as enough large database analysis to be able to bring together a group with diverse scientific backgrounds and areas of expertise.
- MPI structure is well justified.

Weaknesses

- Minor weakness: MPI leadership plan refers to Aim 3a and 3b, but the main proposal does not have an Aim 3a and 3b, just a full Aim 3. So, it is a little difficult to know how the MPI team is going divide up that Aim.

3. Innovation

Strengths

- Use of the federated data model is an innovative approach, and learnings will likely be applicable elsewhere.
- Adding NLP to extract more nuanced clinical data is a strength.

Weaknesses

- None noted by reviewer.

4. Approach.

Strengths

- Robust large database of clinical notes, claims data, and ability to link to ACS and other spatial-temporal datasets, with existing data collection and access infrastructures that the investigators have used before.
- Strong and creative application of NLP and federated data models.
- Plan for internal validation of the NLP model.

Weaknesses

- Major: Unclear how much time is appropriate for the progression subtype assessments—the proposed timeline is 2 years before and after asthma symptom onset, but there is no stated rationale for why this is an appropriate amount of time, or any validation work that has been done for this time period. Given the proposal to have the progression subtypes to be fed into a CDS tool, this issue of data timeline and large potential for missingness is a substantial weakness.
- Major: Unstructured clinical notes, while likely somewhat more sensitive than ICD codes for SDOH, are still a limited source of SDOH data—documentation may vary substantially by

clinician and family. This is a large potential source of data not missing at random and should be more clearly addressed, in the NLP model and also in the progression subtype analysis. While the internal validation of the NLP checks the model against the medical record through chart reviews, there is no check proposed for the model against patient or family reporting, to assess true sensitivity (and specificity, though this is likely of less concern than the missing data) of the model. Note: This is a problem that affects many efforts around use of SDOH and the federal government is beginning to address it by requiring SDOH collection in some quality programs, which creates incentives for structured data fields for these elements in the EHR, as is happening in some EHR systems.

- Major: Similarly, the progression subtype model relies on clinician documentation of exposome and daily symptoms, which also may be poorly documented.
- Moderate: SDOH might change over time, as might disease progression subtype, given the natural progression of asthma, but there is no plan for how to address these time-varying variables in the CDS tool.
- Moderate: There is a fair amount of time and energy on the application developing the CDS tool (prototyping and having people be able to test it), but it is unclear how the data to inform the CDS tool would be generated in real time, so seems like that is putting the cart before the horse and needs an additional step before implementation.
- Minor: SAC membership (policy makers and payers) and areas of input for those members, anticipates ability to assess impact on costs of care, but this work is not far enough along to anticipate this—clinical medicine does not yet have treatment guidelines that would be able to address the progression subtypes.

5. Environment

Strengths

- Strong academic environment with resources and infrastructure able to support this project.

Weaknesses

- Moderate: Clinics are not yet identified for participation and plan for 70 focus group participants, meeting twice, is ambitious given the clinics are not yet identified. This suggests the potential for failure to recruit successfully.

Study Timeline:

Strengths

- Well described and appropriate timeline.

Weaknesses

- None noted by reviewer.

Protections for Human Subjects:

Acceptable Risks and/or Adequate Protections

- Adequate. Rationale given for not getting consent for secondary database. IT security elements in place and federated data approach is designed to allow for minimal risk of data breaches.

Data and Safety Monitoring Plan (Applicable for Clinical Trials Only):

Not Applicable (No Clinical Trials)

Inclusion Plans:

- Sex/Gender: Distribution justified scientifically.
- Race/Ethnicity: Distribution justified scientifically.
- For NIH-Defined Phase III trials, Plans for valid design and analysis: Not applicable.
- Inclusion/Exclusion Based on Age: Distribution justified scientifically.
- All well justified distributions and will likely have diverse representation. There is a higher proportion of male patients than female, but this reflects the asthma population and there is still a large proportion of female patients (40-45%).

Vertebrate Animals:
Not Applicable (No Vertebrate Animals)

Biohazards:
Not Applicable (No Biohazards)

Resource Sharing Plans:
Acceptable

- Robust resource sharing plan, including excellent resources to be shared from the federate learning model as well as a software sharing plan. Data will not be shared, except for potential smaller de-identified sample datasets for use with the open access software.

Budget and Period of Support:
Recommend as Requested

CRITIQUE 3

Significance: 3
Investigator(s): 2
Innovation: 3
Approach: 4
Environment: 1

Overall Impact: In the proposed research privacy-preserving machine learning (ML) tools will be applied to about 20 million patients to identify pediatric asthma subtypes and estimate their progression over time. Improved primary care and emergency treatment of patients with asthma will be facilitated if one correctly identifies the specific asthma subtype. The overall goal is to improve the care and outcomes for millions of such patients. The proposed work is ambitious but not much is said about the new science that will come out from the proposed work.

1. Significance:

Strengths

- Pediatric asthma is a strain, and it severely affects minority children. Absence of asthma subtype characterization prevents better primary and emergency care of patients. Thus, it is important to advance the process of subtyping of asthma.
- Novel ML approaches for estimating pediatric asthma progression including how subtypes/severity change over time. Currently, pediatric asthma progression is poorly understood.
- Novel methods for extracting pediatric asthma patients, their characteristics, and outcomes from real-world data.
- Identification of site-specific and pediatric asthma progression subtypes across OneFlorida+.

2. Investigator(s):

Strengths

- The team is versatile and strong.

Weaknesses

- None noted by reviewer.

3. Innovation:

Strengths

- Creation of a clinical research network to build a real-world data-driven pediatric asthma learning health system.
- Novel federated ML approaches for identifying asthma progression subtype predictive of patients' outcomes.
- Leveraging natural language processing (NLP) to augment asthma computable phenotypes.
- Inclusion of heterogeneous data in the study.

Weaknesses

- No innovations in science are expected (the novelty of the federated ML approaches is not explained).

4. Approach:

Strengths

- Development of computable phenotypes and NLP methods and tools that can extract pediatric asthma patients.
- Modeling of temporal progression pathways of pediatric asthma subtypes with federated ML.
- Identifying site-specific and shared pediatric asthma progression subtypes across OneFlorida+.
- Integration of progression subtype data into the EHR.

Weaknesses

- The title suggests the development of novel privacy-preserving federated machine learning methods. Little is said about the actual novelties of the proposed methods for modeling of temporal progression pathways. It appears that the team will use off-the-shelf methods.
- It is stated that federated learning will be used to enrich and normalize the progression subtypes using the entire OneFlorida+ dataset. It seems that the proposed work is developmental, and that no new science will be produced in this space.

5. Environment:

Strengths

- Strong environment.

Weaknesses

- None noted by reviewer.

Study Timeline:

Strengths

- None noted by reviewer.

Weaknesses

- None noted by reviewer.

Protections for Human Subjects:

Acceptable Risks and/or Adequate Protections

Vertebrate Animals:

Not Applicable (No Vertebrate Animals)

Biohazards:

Not Applicable (No Biohazards)

Budget and Period of Support:

Recommend as Requested

THE FOLLOWING SECTIONS WERE PREPARED BY THE SCIENTIFIC REVIEW OFFICER TO SUMMARIZE THE OUTCOME OF DISCUSSIONS OF THE REVIEW COMMITTEE, OR REVIEWERS' WRITTEN CRITIQUES, ON THE FOLLOWING ISSUES:

PROTECTION OF HUMAN SUBJECTS: ACCEPTABLE

INCLUSION OF WOMEN PLAN: ACCEPTABLE

INCLUSION OF MINORITIES PLAN: ACCEPTABLE

INCLUSION ACROSS THE LIFESPAN: ACCEPTABLE

COMMITTEE BUDGET RECOMMENDATIONS: The budget was recommended as requested.

REVISION NOTE:
Although the application was reviewed in the New Investigator cluster, at the time of application submission, one of the mPIs was mistakenly missing their designation as an early-career investigator (ESI) due to an eRA Commons error. We are re-releasing the summary statement to include the contact PI’s designation as an ESI. The remaining content of the summary statement was not affected by this change.

Footnotes for 1 R01 HL169277-01; PI Name: Fishe, Jennifer Noel

Ad hoc or special section application percentiled against "Total CSR" base.

NIH has modified its policy regarding the receipt of resubmissions (amended applications).See Guide Notice NOT-OD-18-197 at https://grants.nih.gov/grants/guide/notice-files/NOT-OD-18-197.html. The impact/priority score is calculated after discussion of an application by averaging the overall scores (1-9) given by all voting reviewers on the committee and multiplying by 10. The criterion scores are submitted prior to the meeting by the individual reviewers assigned to an application, and are not discussed specifically at the review meeting or calculated into the overall impact score. Some applications also receive a percentile ranking. For details on the review process, see
http://grants.nih.gov/grants/peer_review_process.htm#scoring.
